# Supplementary material for: The impact of spaced learning within physics lessons in secondary schools
Source: PLoS One. 2025 Apr 16;20(4):e0321552. doi: 10.1371/journal.pone.0321552 (PMC12002483; doi:10.1371/journal.pone.0321552)
Supplement: S1 File — (PDF) [file pone.0321552.s001.pdf]

1. This question is about four common nuclear radiations:

alpha, **beta**, **gamma**, **neutron**.

Match each description with **one** of the four radiations.

[4 marks]

Add **one tick** (✓) to **each row** of the table.

You may tick each column once, more than once or not at all.

	Description	alpha	beta	gamma	neutron
1 (a)	has a range of about 50 cm in air				
1 (b)	consists of 2 protons and 2 neutrons				
1 (c)	has the largest mass				
1 (d)	is an electromagnetic wave				

2. What is the typical size of an atom?

[1 mark]

Tick (✓) **one** box.

10^{-3} m ☐

10^{-7} m ☐

10^{-10} m ☐

10^{-12} m ☐

10^{-15} m ☐

3. This question is about the structure of an atom.

3 (a) Which particles can be found in an atomic nucleus?

[1 mark]

Tick (✓) **one** box.

electrons and neutrons ☐

electrons and protons ☐

neutrons and protons ☐

electrons only ☐

neutrons only ☐

3 (b) Complete the sentences. Choose answers from the box.

[1 mark]

**electrons**	**ions**	**neutrons**	**nucleus**	**protons**

The mass of an atom is concentrated in its ___________________.

The mass of the ___________________ is a tiny fraction of the mass of the atom.

3 (c) Complete the table to show the charge of each particle.

[1 mark]

**Name of particle**	**Charge** (positive / negative / uncharged)
**electron**	
**neutron**	
**proton**	

4. Look at the four isotopes below:

$^{10}_{4}\mathrm{Be}$ beryllium-10 $^{11}_{5}\mathrm{B}$ boron-11

$^{9}_{6}\mathrm{C}$ carbon-9 $^{13}_{9}\mathrm{F}$ fluorine-13

4 (a) How many electrons exist in an atom of fluorine-13?

[1 mark]

Number of electrons ___________________________

4 (b) Which of these isotopes has the smallest nuclear mass?

[1 mark]

____

4 (c) Which of these isotopes contains 4 neutrons?

[1 mark]

____

5. Lead-194 has a half-life of 12 minutes.

A sample of lead-194 is prepared.

The lead is left for 36 minutes.

Calculate the fraction of the lead-194 atoms remaining 36 minutes after the sample is prepared?

[2 marks]

____

____

____

____

Fraction remaining = ____________________________

6 (a) The graph below shows the activity of a radioactive sample.

Use the graph to determine the half-life of the sample.

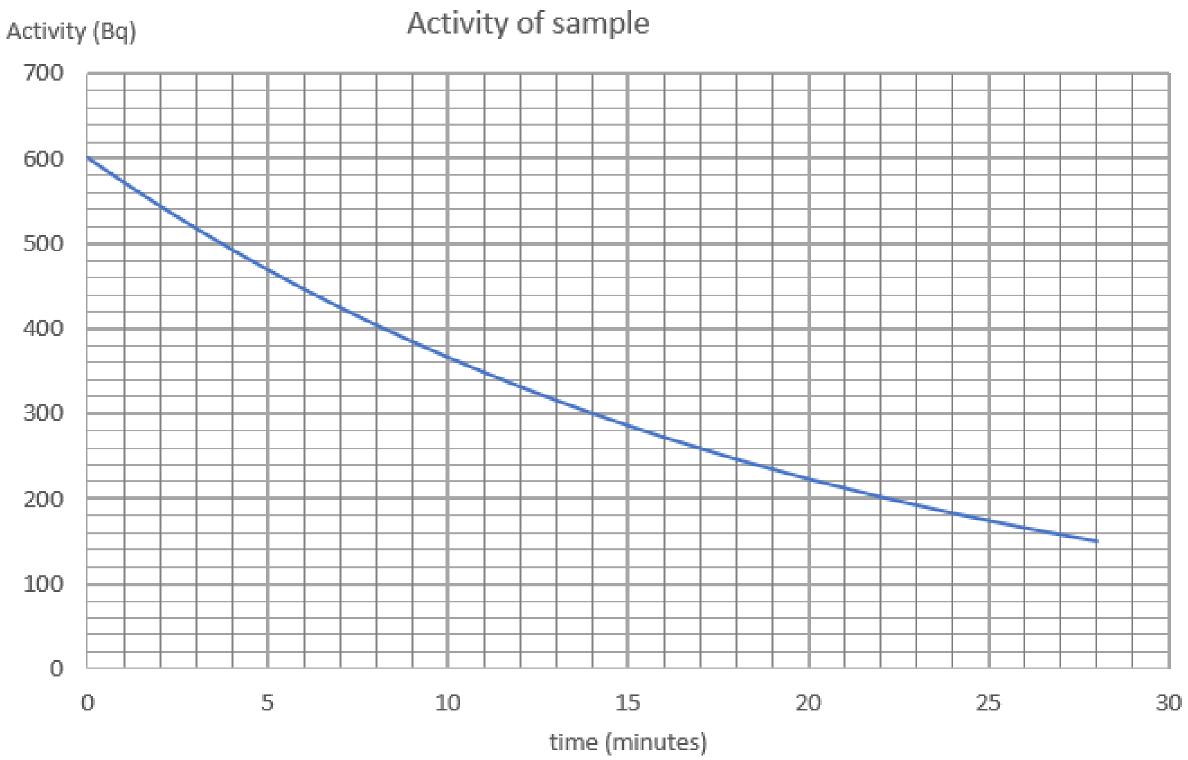

[1 mark]

Half-life = ____________________ minutes

6 (b) A safe way to dispose of this material is: keep the sample for 2 hours and then pour down the sink; rinse the sink with at least 2 litres of tap water.

Suggest why it is considered safe to dispose of this material in this way.

____

____

____

[2 marks]

7. Bismuth-212 decays into polonium-212.

7 (a) Complete the nuclear equation to describe this decay.

[2 marks]

$$^{212}_{83}\text{Bi} \rightarrow\ ^{212}_{84}\text{Po} +\ ^{\square}_{\square}\square$$

7 (b) Describe how this equation shows any changes to the mass and charge of the bismuth nucleus.

[2 marks]

Mass: ____

____

Charge: ____

____

More questions in the next page

8. Atoms and ions may contain protons, neutrons and electrons.

The table below shows information for five particles **A**, **B**, **C**, **D** and **E**.

Each particle (A, B, C, D, E) is an atom or an ion.

Particle	Number of protons	Number of neutrons	Number of electrons
**A**	8	8	7
**B**	8	8	8
**C**	9	8	9
**D**	9	9	9
**E**	10	9	9

8 (a) Which two particles in the table are ions?

[1 mark]

Two ions: particle _____ and particle ______.

8 (b) Which two particles in the table are isotopes of the same element?

[1 mark]

Two isotopes of the same element: particle _____ and particle ______.

More questions in the next page

9. In the early twentieth century, scientists developed their understanding of the atom.

In one experiment, **alpha** radiation was directed at a very thin sheet of gold.

Scientists observed that:

- most alpha particles passed straight through the gold leaf
- a small amount of the alpha particles was deflected by large angles
- a tiny amount of the alpha particles was deflected by more than 90 degrees

Suggest what observations might have been made if **beta** radiation had been used in place of alpha.

Use your knowledge of atomic structure and nuclear radiations to justify your suggestion.

[2 marks]

____

____

____

____

____

More questions in the next page

10. Jo works in a hospital. She works with some radioactive liquids. She has to be careful about **contamination** and **irradiation**.

Compare contamination and irradiation. In your answer:

- state what each term means
- *compare the hazards related to contamination and irradiation*
- suggest one precaution to reduce Jo’s risk when she is working with radioactive liquids.

[6 marks]

____

____

____

____

____

____

____

____

____

____

____

____

____

11. Nuclear fusion reactions take place in stars.

One stage of the process involves hydrogen-1 and hydrogen-2.

Choose two statements that correctly describe this fusion reaction.

[2 marks]

Tick (✓) **two** boxes.

Two hydrogen atoms combine to form a hydrogen molecule.	☐
Two hydrogen atoms combine with an oxygen atom to form H_2O.	☐
Two hydrogen nuclei combine to form helium.	☐
The mass of the products is less than the mass of the reactants.	☐
The mass of the products is more than the mass of the reactants.	☐
The mass of the products is the same as the mass of the reactants.	☐

More questions in the next page

12. Nuclear radiations are used in hospitals.

The table below gives information about some radioactive sources.

**Source**	**Radiation emitted**	**Half-life**
americium-241	alpha	430 years
cobalt-60	gamma	5.3 years
molybdenum-99	beta	2.8 days
radium-223	alpha	11 days
strontium-90	beta	29 years
technetium-99	gamma	6.0 hours

A hospital wants a source of radiation to sterilise surgical equipment such as metal scissors and scalpels.

Suggest **one** source that might be suitable. Justify your choice using information from the table.

[4 marks]

____

____

____

____

____

____

____

____

____

13. In nuclear power stations, uranium-235 can be used in fission reactions.

13 (a) Describe the process of nuclear fission and how a chain reaction is established.

[3 marks]

____

____

____

____

____

____

____

____

13 (b) The chain reaction must be carefully controlled to produce electricity safely.

Describe how the chain reaction is controlled.

[1 mark]

____

____

End of questions
